# Handgrip Strength and Muscle Mass Indices in the Assessment of Muscle Strength and Muscle Mass Parameters in Women Aged 65–75 Years with Low Physical Activity

**DOI:** 10.3390/nu18050795

**Published:** 2026-02-28

**Authors:** Karolina Klimek, Agnieszka Gdańska, Tomasz Jurys, Ewa Sadowska-Krępa, Mateusz Grajek

**Affiliations:** 1 Department of Economics and Health Care Management, Faculty of Public Health in Bytom, Medical University of Silesia in Katowice, 40007 Katowice, Poland; ksobczyk@sum.edu.pl; 2 Department of Public Health, Faculty of Public Health in Bytom, Medical University of Silesia in Katowice, Piekarska 18, 41902 Bytom, Poland; a.gdanska@o2.pl; 3 Department of Rehabilitation, Faculty of Health Sciences in Katowice, Medical University of Silesia in Katowice, Medyków 12, 40751 Katowice, Poland; tomasz.jurys@sum.edu.pl; 4 Institute of Sport Sciences, The Jerzy Kukuczka Academy of Physical Education, 40-065 Katowice, Poland; e.sadowska-krepa@awf.katowice.pl

**Keywords:** sarcopenia, handgrip strength, body composition, skeletal muscle index, older women, physical activity, sarcopenic obesity, aging, nutritional assessment

## Abstract

Background: Age-related alterations in body composition, including the decline of skeletal muscle mass and strength, combined with increased adiposity, contribute to sarcopenia risk in older adults. Handgrip strength (HGS) is widely used as a functional marker of muscle health. Objective: To evaluate the associations between body composition, handgrip strength, and step-based physical activity in women aged 65–75 years. Material and Methods: A cross-sectional observational study was conducted in 246 community-dwelling women. Body composition, including Skeletal Muscle Index (SMI), was assessed using bioelectrical impedance analysis. Handgrip strength was measured with dynamometry according to the Southampton protocol. Physical activity was evaluated using pedometers. Group comparisons and correlation analyses were performed. Results: Overweight or obesity was present in 75% of the participants. Reduced SMI was observed in 62.1% of women, while low HGS (<20 kg) occurred in 78.0%. Women with normal SMI demonstrated significantly higher HGS values. HGS correlated positively with lean body mass and SMI but not with BMI. Participants achieving ≥ 5000 steps/day showed significantly higher muscle mass indices and strength. Conclusion: Unfavorable body composition and reduced muscle strength were highly prevalent. HGS was strongly associated with muscle mass parameters, supporting its role as a functional marker independent of BMI. Step-based activity was associated with more favorable muscle-related outcomes.

## 1. Background

It is estimated that over the next 30 years, demographic trends will shift, leading to a substantial increase in the proportion of older adults in society. This change will result from the observed extension of average life expectancy in recent decades [1]. Population ageing constitutes a major factor generating significant social consequences, particularly related to the need to adapt healthcare systems to the specific needs of older adults. Due to age-related changes, seniors are at increased risk of developing multiple chronic diseases, which in turn leads to a growing demand for health and long-term care services [2,3].

The ageing process is associated with numerous changes in bodily functioning observed at biological, psychological, and social levels. Age-related processes result in an increased risk of disease, functional decline, and reduced quality of life [2,4]. Diseases whose risk increases with age include cardiovascular diseases, respiratory diseases, cancers, diabetes, overweight and obesity, mental disorders, and neurodegenerative diseases. These conditions represent one of the leading causes of mortality among the global older adult population [3]. In addition, ageing is accompanied by the occurrence of so-called age-related diseases. These conditions arise from natural ageing processes and encompass a wide range of multisystem disorders [4]. This group includes overweight and obesity associated with a reduced metabolic rate, osteoporosis resulting from decreased bone mass and density, and musculoskeletal disorders that impair mobility. A decline in muscle mass may lead to sarcopenia, while in older adults with concomitantly high levels of adipose tissue, it increases the risk of obesity [5].

In obesity, high body mass relative to the diagnostic BMI and an excessive proportion of body fat are observed, defined as >35% in women and >30% in men [6]. Overweight and obesity among older adults constitute one of the greatest challenges for healthcare systems worldwide in both developed and developing countries, as obesity increases mortality risk and may lead to the development of multiple chronic diseases [2]. In obese individuals, a condition known as sarcopenic obesity is frequently observed. This condition is characterized by excessive fat mass accompanied by a loss of muscle mass, resulting in reduced muscle strength and impaired skeletal muscle function. Sarcopenic obesity is particularly dangerous in older adults, as high body weight may substantially hinder the detection of muscle mass loss. Nutritional status assessment based solely on body weight measurement, without body composition analysis, does not provide sufficient information on muscle mass in seniors and may delay diagnosis [7,8].

The reduction in muscle mass characteristic of sarcopenia is diagnosed based on the presence of three components: low muscle strength, low muscle quantity or quality, and poor physical performance [9]. Sarcopenia is a generalized and progressive muscle disorder that leads to numerous adverse outcomes in older adults, including falls, fractures, bone fragility, reduced ability to perform activities of daily living, and even increased mortality risk [7,8]. The presence of sarcopenia increases the likelihood of disability and hospitalization among older individuals. Loss of muscle mass results in reduced muscle strength, which is crucial for maintaining daily functional abilities such as walking and lifting, as well as overall physical capacity [10]. Reduced muscle strength adversely affects daily functioning, physical performance, independence, and quality of life [8,11]. Consequently, low muscle strength is considered an indicator of increased risk of cardiovascular diseases, physical disability, and mortality.

Among the methods used to assess muscle strength in older adults, handgrip strength (HGS) measurement is commonly applied. Handgrip strength is an effective tool for assessing the risk of sarcopenia or osteoporosis and enables monitoring of the ageing process [10,12].

Delaying the onset of sarcopenia or reducing its consequences can be achieved primarily through lifestyle modifications, particularly dietary changes (increased protein intake) and physical activity. Its development is influenced not only by coexisting risk factors but also by genetic and lifestyle-related factors acting throughout the life course. Physical activity plays a particularly important role, as the decline in muscle mass and the reduced capacity for muscle synthesis begin after the age of 40 and progress into old age [9,11]. Therefore, lifestyle modification involving dietary changes, improved nutritional quality, optimal protein intake, and regular physical exercise is recommended as the most effective intervention for preventing sarcopenia [11,13]. Sarcopenia has become a subject of intensive research, as currently there are no pharmacological treatments approved for its management in older adults other than physical activity and dietary interventions [13].

Nutritional factors play a fundamental role in the pathogenesis and progression of sarcopenia. Aging is associated with physiological changes that negatively affect dietary intake, digestion, absorption, and metabolic utilization of nutrients, thereby increasing the risk of protein–energy malnutrition even in individuals with excessive body mass. In particular, insufficient protein intake and suboptimal consumption of essential amino acids contribute to impaired muscle protein synthesis and accelerated loss of skeletal muscle mass. In addition, deficiencies in key micronutrients, especially vitamin D, have been linked to reduced muscle strength, impaired neuromuscular function, and a higher risk of falls in older adults [9]. Contemporary evidence indicates that adequate protein intake, appropriate dietary quality, and nutritional screening should be considered integral components of sarcopenia prevention and management strategies, alongside physical activity interventions [14].

Understanding the pathophysiology of sarcopenia and developing effective diagnostic methods are essential for designing successful preventive strategies. Current research in the field of older adult population health indicates that the diagnosis and prevention of sarcopenia are likely to become an integral part of clinical practice in the near future [9]. Both obesity and sarcopenia contribute to increased mortality risk, a higher prevalence of chronic diseases, and deterioration of functional capacity. Additionally, they reduce quality of life and represent a major challenge for healthcare systems worldwide [4]. In light of these considerations, further research is necessary to identify the most effective strategies for preventing these disorders in the older adult population.

The present study addresses the need for integrated evaluation of structural and functional muscle parameters in older women, focusing on the relationships between skeletal muscle index, handgrip strength, and objectively measured ambulatory activity. This approach reflects contemporary sarcopenia frameworks emphasizing the combined assessment of muscle quantity and muscle function.

Accordingly, the aim of the present study was to assess body mass and body composition, physical activity, and handgrip strength in women aged 65–75 years characterized by predominantly sedentary or low levels of ambulatory physical activity.

## 2. Materials and Methods

### 2.1. Study Participants

The study group consisted of 264 women aged 65–75 years who were classified as overweight or obese and who participated in activities organized by Universities of the Third Age and Active Senior Clubs in the Silesian Voivodeship (Poland). The exclusion criteria included age < 65 years or >75 years, diseases requiring a specialist diet, and conditions that could limit daily physical activity, such as musculoskeletal disorders, cardiovascular diseases, diabetes, and hypertension. Initially, 264 women were recruited. Eighteen participants were excluded due to incomplete datasets or failure to meet measurement criteria. The final analysis included 246 women.

Prior to enrollment, all participants underwent a medical consultation to exclude contraindications to participation in the study. Participation was voluntary. The participants were informed about the aim and procedures of the study and about their right to withdraw at any stage without consequences.

Participants were not recruited based on BMI category. Body mass status was evaluated post hoc using the WHO BMI criteria. The study sample, therefore, includes women across BMI categories, allowing analysis of associations independent of weight status.

### 2.2. Anthropometric Measurements

Anthropometric measurements were performed in all participants and included body height, body weight, waist circumference, and hip circumference. The following body composition and anthropometric indicators were assessed:

- Fat mass (BF, kg) and lean body mass (LBM, kg).
- Percentage fat mass (%BF) and percentage lean body mass (%LBM), measured using bioelectrical impedance analysis (BIA) with a Tanita TBF-300M analyzer.
- Body mass index (BMI) and waist-to-hip ratio (WHR), calculated using the following reference values: BMI < 18.5—underweight; 18.5–24.9—normal weight; 25.0–29.9—overweight; ≥30.0—obesity and WHR ≥ 0.80. WHR was calculated as waist circumference divided by hip circumference [1].

### 2.3. Assessment of Sarcopenia

Sarcopenia was assessed using the following measures:

- Skeletal Muscle Index (SMI) is a commonly used indicator in sarcopenia diagnostics that evaluates skeletal muscle mass relative to height or body mass. An SMI value below the established cut-off (<5.5 kg/m^2^ in women, according to EWGSOP) indicates a high risk of sarcopenia associated with reduced muscle mass and strength. Skeletal Muscle Index (SMI) was calculated as appendicular lean mass divided by height squared (kg/m^2^). SMI was measured using BIA (Tanita TBF-300M, Tokyo, Japan). BIA measurements were performed under standardized conditions. Participants were measured in the morning, in a fasted state, after an overnight rest, and were instructed to avoid vigorous physical activity and excessive fluid intake for 12 h prior to assessment. Measurements were conducted according to manufacturer guidelines to minimize hydration-related variability.
- Handgrip strength (HGS). HGS was measured using a professional dynamometer (MG 4800, Charder: Taichung City, Taiwan, China), which allows assessment of maximal handgrip strength expressed as the mean of several trials. To ensure accuracy and repeatability, measurements were conducted according to the standardized Southampton protocol, which is widely applied and minimizes the influence of participant positioning on the results. Adequate rest intervals between trials were maintained to avoid fatigue (Table 1).

### 2.4. Physical Activity

Physical activity was assessed using an objective measurement tool—pedometers—manufactured by Yamax Inc. (Kumamoto, Japan). The following step-count-based thresholds proposed by Oliveira et al. (2019) were adopted: <5000 steps/day—sedentary lifestyle; 5000–7499 steps/day—low physical activity; 7500–9999 steps/day—moderate physical activity; and ≥10,000 steps/day—recommended (desirable) physical activity level [15]. The participants were instructed on the proper use of the pedometers. The pedometers recorded the total number of steps accumulated over one week. Devices were removed only during bathing and sleeping. Daily step count was interpreted as an indicator of ambulatory behavior rather than total physical activity.

### 2.5. Ethics

The study was conducted in accordance with the Declaration of Helsinki and the principles of Good Clinical Practice. All participants were fully informed about the study objectives and procedures and provided written informed consent prior to participation. Anonymity and confidentiality were strictly maintained, and participants were free to withdraw from the study at any stage without providing a reason. This study was conducted in accordance with the Ethics Committee of the Medical University of Silesia in Katowice (decision: PCN/CBN/0052/KB/187/22; 12 July 2022).

All participants received a written information sheet detailing the purpose of the study. Recruitment was conducted directly through a women’s association meeting at a Senior Club in Rybnik, where participants regularly engaged in various recreational activities. All participants were instructed not to modify their usual daily behavior patterns and not to undertake additional physical exercise during the study period in order to avoid potential bias and preserve the validity of the results.

### 2.6. Statistical Analysis

Statistical analyses were performed using standard descriptive and inferential statistical methods appropriate to the data characteristics and study objectives. For all quantitative variables, measures of central tendency and variability were calculated, including the arithmetic mean, standard deviation, median, and minimum and maximum values. The normality of data distribution was assessed using the Shapiro–Wilk test, which is recommended for biomedical data analysis due to its high statistical power. Homogeneity of variances between comparison groups was verified using Levene’s test.

Comparisons between two independent groups (e.g., women with BMI < 25 kg/m^2^ vs. ≥25 kg/m^2^; normal vs. reduced SMI; physical activity < 5000 vs. ≥5000 steps/day) were performed using the independent-samples Student’s *t*-test when assumptions of normality and homogeneity of variance were met. When these assumptions were violated, the nonparametric Mann–Whitney U test was applied. For categorical variables, such as BMI categories, SMI status, or physical activity levels, frequencies and percentages were compared using the chi-square (χ^2^) test, and Fisher’s exact test was employed when expected cell counts were small.

Associations between quantitative variables were evaluated using correlation analysis. Pearson’s linear correlation coefficients were calculated for variables with approximately normal distributions, whereas Spearman’s rank correlation coefficients were used for variables that did not meet the normality criterion. The strength and direction of associations were interpreted in accordance with commonly accepted criteria in the literature, taking into account both the magnitude of the correlation coefficient and the level of statistical significance.

Additionally, stratified analyses were conducted to assess differences in SMI and HGS values according to participants’ occupational characteristics (employment status and type of work performed), allowing for control of the potential influence of long-term physical workload on muscle-related parameters. Depending on the fulfillment of statistical assumptions, either parametric or nonparametric tests were applied in these analyses.

In all analyses, a two-sided significance level of α = 0.05 was adopted, and *p*-values < 0.05 were considered statistically significant. Statistical computations were performed using specialized statistical software designed for biomedical research. Results were presented in tabular and descriptive form, in accordance with principles of clarity and rigorous statistical reporting.

## 3. Results

Data from 246 women aged 65–75 years were included in the statistical analysis. The mean age of the participants was 67.97 ± 2.46 years, covering the full range of the predefined age criterion. The mean body weight was 78.20 ± 12.44 kg, and the mean height was 1.68 ± 0.07 m. Based on these values, the mean body mass index (BMI) was calculated as 29.86 ± 4.34 kg/m^2^. BMI values ranged from 19.0 to 48.0 kg/m^2^. According to the adopted BMI classification criteria, overweight or obesity (BMI ≥ 25 kg/m^2^) was identified in 75.0% of the participants (n = 185), whereas BMI values < 25 kg/m^2^ were observed in 25.0% of the women (n = 61).

The mean waist-to-hip ratio (WHR) was 0.87 ± 0.12, with values ≥ 0.80—indicative of abdominal obesity—recorded in more than 70% of the participants. The mean percentage of body fat (%BF) was 37.25 ± 7.20%, and the mean absolute fat mass was 30.82 ± 8.46 kg. %BF values exceeding 35% were observed in the majority of the participants, while values below this threshold were recorded in less than one third of the women. The mean percentage of lean body mass (%LBM) was 59.91 ± 9.72%, and the mean absolute lean body mass (LBM) was 63.99 ± 12.16 kg, with values ranging from 34.0 to 88.0 kg (Table 2).

Based on bioelectrical impedance measurements, the skeletal muscle index (SMI) was calculated. The mean SMI value was 9.36 ± 2.22 kg/m^2^, with a median of 9.68 kg/m^2^ and a range of 2.0–13.6 kg/m^2^. Reduced SMI values (below the cut-off points adopted for women) were identified in 62.1% of the participants (n = 153), whereas values within the normal range were observed in 37.9% of women (n = 93). Women with BMI < 25 kg/m^2^ exhibited a significantly higher mean SMI (10.8 ± 2.0 kg/m^2^) compared with women with BMI ≥ 25 kg/m^2^ (8.9 ± 2.1 kg/m^2^); this difference was statistically significant (*p* < 0.001).

The mean handgrip strength (HGS) in the entire group was 19.9 ± 4.7 kg, with a median of 19.5 kg and a range from 4.6 to 32.1 kg. HGS values < 20 kg were observed in 78.0% of the participants (n = 192), whereas values ≥ 20 kg were recorded in 22.0% of women (n = 54). Women with normal SMI achieved significantly higher HGS values (22.4 ± 4.1 kg) compared with those with reduced SMI (18.1 ± 4.3 kg); this difference was statistically significant (*p* < 0.001). Similarly, women with BMI < 25 kg/m^2^ demonstrated higher mean handgrip strength (21.9 ± 4.3 kg) than women with BMI ≥ 25 kg/m^2^ (19.2 ± 4.6 kg), with statistical significance at *p* < 0.001. Based on EWGSOP2 criteria (low HGS + low SMI), 45.5% of participants met the criteria for confirmed sarcopenia.

Analysis of physical activity levels showed that 65.2% of the participants (n = 160) did not reach the threshold of 5000 steps per day, whereas 34.8% of women (n = 86) recorded ≥5000 steps/day. Women achieving ≥ 5000 steps/day were characterized by a higher mean SMI (10.1 ± 2.1 kg/m^2^) compared with those performing <5000 steps/day (8.9 ± 2.2 kg/m^2^); this difference was statistically significant (*p* = 0.014). Similarly, mean HGS was higher in the more physically active group (21.0 ± 4.6 kg) than in the less active group (19.1 ± 4.6 kg).

Correlation analysis revealed a strong positive association between lean body mass (LBM) and SMI (r = 0.648; *p* < 0.001). The correlation between body weight and SMI was moderate (r = 0.398; *p* < 0.01), whereas the relationship between BMI and SMI was weak and did not demonstrate clear statistical significance. No significant correlation was found between percentage body fat (%BF) and SMI (*p* > 0.05).

Handgrip strength showed strong positive correlations with LBM (r = 0.595; *p* < 0.001) and SMI (r = 0.545; *p* < 0.001). The association between HGS and step count was weaker but statistically significant (r = 0.249; *p* < 0.05). No statistically significant correlations were observed between HGS and BMI or body weight (*p* > 0.05).

Comparative analyses related to occupational activity revealed no significant differences in SMI (9.4 ± 2.2 vs. 9.2 ± 2.3 kg/m^2^) or HGS (20.0 ± 4.7 vs. 19.6 ± 4.5 kg) between professionally active and inactive women (*p* > 0.05). Women who reported performing manual work achieved higher mean HGS values (20.8 ± 4.8 kg) compared with those engaged in sedentary work (19.4 ± 4.5 kg); however, this difference did not reach statistical significance (*p* = 0.061). Differences in SMI values between these groups were also not statistically significant (Table 3).

## 4. Discussion

The results of this cross-sectional observational study indicate a high prevalence of unfavorable changes in body composition and muscle strength among women aged 65–75 years, characterized by predominantly sedentary or low levels of ambulatory physical activity. This study evaluates parameters associated with sarcopenia risk rather than providing a formal EWGSOP2 diagnosis. Confirmed sarcopenia was operationally defined as coexisting low muscle strength and reduced muscle mass. The findings confirm that the ageing process—particularly when combined with overweight or obesity and insufficient physical activity—promotes the coexistence of reduced muscle mass, low muscle strength, and excessive adipose tissue, a pattern consistent with so-called sarcopenic obesity [7,8,9].

In the studied population, as many as 75% of women were classified as overweight or obese based on BMI, and more than 70% met the criteria for abdominal obesity according to WHR. At the same time, the mean percentage of body fat exceeded 37%, which is clearly above the recommended reference values for older women [6]. These results are consistent with meta-analytic evidence indicating a systematic increase in obesity prevalence among older adults worldwide, particularly in developed countries [2,6]. Moreover, the lack of a significant correlation between BMI and SMI in the present study confirms the limited usefulness of BMI as a standalone diagnostic tool for assessing nutritional status and sarcopenia risk in older individuals, a limitation previously highlighted in population-based studies [7,8].

A particularly important finding of this study is the high proportion of women with reduced skeletal muscle index. More than 62% of participants met the criteria for low SMI according to the EWGSOP cut-off points, despite mean body weight and BMI values frequently falling within the overweight or obese range. This phenomenon is characteristic of sarcopenic obesity, in which excessive fat mass masks the loss of muscle tissue [8,9]. Similar proportions of low muscle mass among overweight older women were reported in the Health, Aging and Body Composition Study, where reduced muscle mass was strongly associated with impaired physical performance and an increased risk of adverse health outcomes [7].

Handgrip strength analysis revealed that as many as 78% of the women achieved HGS values below 20 kg, indicating a marked reduction in muscle strength. These findings are consistent with current literature demonstrating that HGS is one of the most sensitive and prognostically relevant indicators of muscle function in older adults [12]. Cruz-Jentoft et al. (2019) emphasize that low muscle strength represents the first and key criterion for the diagnosis of sarcopenia, often preceding measurable declines in muscle mass [9]. This concept is supported by the present findings, as women with reduced SMI exhibited significantly lower HGS values compared with those with normal SMI.

The strong positive correlations observed between HGS and both LBM and SMI further support the functional value of handgrip strength as a surrogate marker of muscle quantity and quality. Similar associations have been reported in review and experimental studies, where HGS correlated not only with muscle mass but also with functional performance and the risk of hospitalization and mortality [7,10,12]. The absence of a significant correlation between HGS and BMI in this study further underscores that body mass alone does not reflect muscle functional capacity in older adults.

Importantly, impedance-derived parameters provide insight into the qualitative dimension of muscle tissue. Phase angle, reflecting the relationship between resistance and reactance, is widely interpreted as a surrogate marker of cellular membrane integrity and intracellular/extracellular water distribution. Lower phase angle values commonly observed in sarcopenic and sarcopenic-obese individuals suggest deterioration of muscle cell function and structural integrity. Vector displacement analysis further supports the notion that alterations captured by BIA extend beyond simple estimates of muscle mass and may reflect tissue quality and hydration abnormalities [16,17].

Physical activity emerged as another key factor influencing muscle-related outcomes. The majority of participants did not reach the threshold of 5000 steps per day, classifying them as leading a sedentary lifestyle [15]. More physically active women demonstrated significantly higher SMI and HGS values, in full agreement with findings from intervention studies and meta-analyses indicating that even moderate levels of daily physical activity may slow the decline in muscle mass and strength in older adults [11,18]. Shen et al. (2023) showed that regular resistance and multicomponent exercise constitute the most effective non-pharmacological strategy for the prevention and treatment of sarcopenia [11], which is consistent with the observations of the present study.

The lack of significant differences in SMI and HGS between professionally active and inactive women suggests that previous occupational activity alone does not provide sufficient protection in later life. However, the tendency toward higher HGS values among women who reported performing manual work, although not statistically significant, is consistent with the hypothesis of a long-term protective effect of cumulative muscle loading on muscle strength in old age [9,11].

Age-related vector shifts have been consistently reported even among apparently healthy individuals, emphasizing the necessity of age-specific reference ellipses for meaningful interpretation of bioelectrical data. Failure to account for age-dependent physiological changes may result in misclassification or underestimation of clinically relevant muscle impairment, particularly in populations with elevated adiposity where BMI remains insensitive to muscle quality alterations [19].

Nutritional factors represent an important contextual determinant of muscle health in older adults. Age-related anabolic resistance, insufficient protein intake, and micronutrient deficiencies may contribute to impaired muscle protein synthesis and reduced muscle function. Although dietary intake was not directly assessed in this study, these mechanisms provide a relevant physiological background for interpreting muscle mass and strength alterations observed in ageing populations [16,17,19].

The present findings should be interpreted within a broader public health context, in which obesity and sarcopenia are recognized as key determinants of disability, loss of independence, and increased demand for healthcare services in ageing populations [2,3,4]. The high prevalence of low SMI and low HGS observed in this cohort highlights the urgent need to implement integrated preventive strategies that include both body composition assessment and routine handgrip strength measurement in clinical and community settings.

In summary, the results of this study confirm that women aged 65–75 years with low levels of physical activity exhibit an unfavorable coexistence of excessive fat mass, reduced muscle mass, and low muscle strength. Bioelectrical impedance vector analysis (BIVA) studies have demonstrated characteristic vector displacement patterns in individuals with sarcopenia and sarcopenic obesity, typically showing rightward vector migration associated with reduced soft tissue mass and altered hydration status. Furthermore, reduced phase angle values observed in these populations are interpreted as indicators of impaired cellular integrity and diminished muscle quality rather than merely reduced muscle quantity [16,17,19]. This pattern is consistent with current scientific evidence and underscores the importance of early identification of sarcopenia and sarcopenic obesity as major public health challenges in ageing societies. Further research, including longitudinal and interventional studies, is necessary to identify the most effective strategies for preventing muscle mass and strength loss in this population group.

The main strengths of this study include a relatively large sample size for a cross-sectional observational study and a homogeneous group with respect to sex and age, which enhances internal consistency and limits the influence of confounding factors related to sex differences. The use of objective measurement methods—bioelectrical impedance analysis for body composition, handgrip dynamometry performed according to the Southampton protocol, and pedometers for physical activity assessment—constitutes a significant methodological advantage and increases the reliability of the results. An additional strength is the simultaneous analysis of structural (SMI, LBM, %BF) and functional (HGS) parameters, in line with current diagnostic recommendations for sarcopenia, allowing for a more comprehensive assessment of muscle health.

The study also has several limitations. Its cross-sectional design precludes causal inference regarding the relationships between physical activity, body composition, and muscle strength. Although widely used in population studies, BIA has limited precision compared with reference methods such as DXA, particularly in individuals with high levels of body fat. Another limitation is the lack of detailed dietary assessment, including protein intake, which is an important modulator of muscle mass and function in older adults. Although BIA is widely used in epidemiological research, its estimates may be influenced by hydration status and body fat distribution. Therefore, muscle mass values should be interpreted cautiously. Step count reflects ambulatory volume but does not capture physical activity intensity or resistance-type exercise, which are particularly relevant for muscle mass and strength. Finally, the study sample consisted exclusively of women participating in senior clubs and Universities of the Third Age, which may limit the generalizability of the findings to the broader population of older women. Multiple comparisons were performed without formal correction; results should therefore be interpreted cautiously.

## 5. Conclusions

The conducted study demonstrated that women aged 65–75 years characterized by predominantly sedentary or low levels of ambulatory physical activity commonly exhibit unfavorable changes in body composition, including a high proportion of body fat, reduced muscle mass, and low muscle strength as assessed by handgrip strength. The findings confirm the limited usefulness of BMI as a standalone tool for assessing sarcopenia risk and indicate the need to routinely incorporate body composition measurements and functional indicators of muscle strength into health assessments of older adults. It was shown that a higher level of daily physical activity, even of moderate intensity, is associated with a more favorable muscle mass profile and greater handgrip strength, underscoring the importance of physical activity as a key component of sarcopenia prevention. The results highlight the need for early identification of individuals at risk of sarcopenia and sarcopenic obesity, as well as the implementation of integrated preventive strategies that include regular physical activity and monitoring of nutritional status in the population of older women.

## Figures and Tables

**Table 1:** Rest intervals between HGS trials.

Age	P_5_	P_10_	P_20_	P_30_	P_40_	P_50_	P_60_	P_70_	P_80_	P_90_	P_95_
65–69	14.3	16.6	19.5	21.6	23.3	25.0	26.6	28.4	30.5	33.4	35.8
70–74	13.2	15.5	18.3	20.3	22.0	23.6	25.2	26.9	28.9	31.8	34.1
75–79	12.0	14.3	17.0	18.9	20.5	22.1	23.6	25.2	27.2	29.9	32.2

**Table 2:** Group characteristics (N = 246).

Variable	Control
Age (years)	67.97 ± 2.46
Weight (kg)	78.20 ± 12.44
Height (m)	1.68 ± 0.07
BMI (kg/m^2^)	29.86 ± 4.34
WHR	0.87 ± 0.12
%BF	37.25 ± 7.2
BF (kg)	30.82 ± 8.46
%LBM	59.91 ± 9.72
LBM (kg)	63.99 ± 12.16

BMI—Body Mass Index; WHR—Waist-to-Hip Ratio; BF—Body Fat; LBM—Lean Body Mass.

**Table 3:** Associations between body composition indices, handgrip strength, physical activity, and occupational characteristics in older women (N = 246).

Variable	Category	n	%	SMI (kg/m^2^), Mean ± SD	HGS (kg), Mean ± SD	*p* Value
BMI	<25 kg/m^2^	61	25.0	10.8 ± 2.0	21.9 ± 4.3	<0.001
	≥25 kg/m^2^	185	75.0	8.9 ± 2.1	19.2 ± 4.6	
SMI	Normal SMI	93	37.9	–	22.4 ± 4.1	<0.001
	Low SMI	153	62.1	–	18.1 ± 4.3	
HGS	≥20 kg	54	22.0	11.2 ± 1.9	–	<0.001
	<20 kg	192	78.0	8.7 ± 2.0	–	
Steps	≥5000	86	34.8	10.1 ± 2.1	21.0 ± 4.6	0.014
	<5000	160	65.2	8.9 ± 2.2	19.1 ± 4.6	
Occupational	Previously employed	189	76.8	9.4 ± 2.2	20.0 ± 4.7	>0.05
	Not employed	57	23.2	9.2 ± 2.3	19.6 ± 4.5	
Type of work	Manual work	83	33.7	9.7 ± 2.3	20.8 ± 4.8	0.061
	Sedentary work	163	66.3	9.2 ± 2.2	19.4 ± 4.5	

BMI—Body Mass Index; SMI—Skeletal Muscle Index; HGS—Handgrip Strength.

## Data Availability

Data is maintained within this article.
